# Bilateral Symmetry Has No Effect on Stereoscopic Shape Judgments

**DOI:** 10.1177/20416695211042644

**Published:** 2021-08-31

**Authors:** Ying Yu, Alexander A. Petrov, James T. Todd

**Affiliations:** Department of Psychology, Ohio State University, Columbus, Ohio, United States

**Keywords:** 3D perception, shapes/objects, shape, stereopsis

## Abstract

A single experiment is reported that measured the apparent stereoscopic shapes of symmetric and asymmetric objects at different viewing distances. The symmetric stimuli were specifically designed to satisfy the minimal conditions for computing veridical shape from symmetry. That is to say, they depicted complex, bilaterally symmetric, plane-faced polyhedra whose symmetry planes were oriented at an angle of 45° relative to the line of sight. The asymmetric stimuli were distorted versions of the symmetric ones in which the 3D position of each vertex was randomly displaced. Prior theoretical analyses have shown that it is mathematically possible to compute the 3D shapes of symmetric stimuli under these conditions, but those algorithms are useless for asymmetric objects. The results revealed that the apparent shapes of both types of objects were expanded or compressed in depth as a function of viewing distance, in exactly the same way as has been reported in many other studies, and that the presence or absence of symmetry had no detectable effect on performance.

One of the fundamental problems of human perception is the need to recognize objects over a wide range of viewing conditions. The optical projections of objects that provide us with visual information can change dramatically as a function of viewing distance, orientation, or the pattern of illumination, yet our perceptions of 3D structure remain relatively stable over these changes—what is often referred to as shape constancy. However, our ability to achieve shape constancy is far from perfect, and measurable distortions of apparent shape have been reported for several different sources of optical information, such as shading, texture, motion, or binocular disparity.

For example, one well-documented violation of shape constancy occurs when objects are viewed stereoscopically at different distances, such that more distant objects appear compressed in depth relative to physically identical ones that are closer to the observer (e.g., Baird & Biersdorf, 1967; Campagnoli et al., 2017; Campagnoli & Domini, 2019; Gilinsky, 1951; Glennerster et al., 1996, 1998; Harway, 1963; Hecht et al., 1999; Heine, 1900; Johnston, 1991; Johnston et al., 1994; Norman et al., 1996; Scarfe & Hibbard, 2006; Shaffer et al., 2008; Todd & Norman, 2003; Toye, 1986; Wagner, 1985). This occurs because the disparity difference between any two visible points changes systematically with viewing distance (Howard & Rogers, 2002). Thus, in order to obtain an accurate estimate of 3D structure from binocular disparity, it is necessary to somehow compensate for variations in viewing distance, and the empirical evidence indicates that human observers are unable to do that accurately.

One possible way of solving this disparity scaling problem is to combine binocular disparity with other sources of information (Johnston et al., 1994; Richards, 1985; Todd & Norman, 2003). For example, researchers in computer vision have shown that when a visible object is bilaterally symmetric, this fact imposes important constraints on its possible 3D interpretations (see Gordon, 1989; Jayadevan et al., 2017; Michaux et al., 2017; Yang et al., 2005). Li et al. (2011) have developed a model that can combine symmetry with binocular disparity to compute veridical estimates of 3D shape. It is also important to note, however, that there are some minimal conditions that must be satisfied for the application of this model. In particular, the depicted object must be a bilaterally symmetric, plane-faced polyhedron with at least four visible pairs of corresponding points, and the object must be oriented so that the plane of 3D bilateral symmetry is neither parallel nor perpendicular to the observer’s line of sight (see also Li et al., 2009; Vetter & Poggio, 1994).

There is some empirical evidence that stereoscopic shape discrimination for objects presented at different orientations in depth is more accurate for symmetric objects than for asymmetric ones (Chan et al., 2006; Lee & Saunders, 2013). One possible reason for this is that the symmetry plane facilitates comparison across different views by providing a perceptually salient frame of reference. This is supported by Lee and Saunders’ finding that the difference in discrimination accuracy between symmetric and asymmetric objects is reduced by removing uncertainty about the angular difference between the two objects to be compared. A similar result has also been reported by Egan et al. (2012) for monocular shape discrimination. They too found that symmetric objects are judged more accurately than asymmetric ones, but that this effect is eliminated if the objects are bisected by a visible contour to provide a frame of reference for assessing their 3D orientations.

Note that none of these studies manipulated the relative viewing distances of the stimulus objects, so they cannot shed any light on the possible role of symmetry for solving the disparity scaling problem. Although several experiments have used bilaterally symmetric objects to investigate stereoscopic depth scaling (e.g., Glennerster et al., 1996, 1998; Johnston, 1991; Johnston et al., 1994; Todd & Norman, 2003), they did not satisfy the minimal conditions of Li et al.’s model for computing veridical shape from symmetry. Instead, they used relatively simple stimuli with fewer than four visible pairs of corresponding points and/or the objects were oriented so that their 3D symmetry plane was nearly parallel or perpendicular to the observer’s line of sight.

The experiments of Li et al. (2011), in contrast, used stimuli that did satisfy these conditions, and they obtained veridical shape matching performance at different viewing distances. However, the adjustment task they employed did not allow observers to indicate whether or not the test objects appeared expanded or compressed in depth, which is the main type of distortion found in previous investigations. In another relevant experiment from the same lab, Jayadevan et al. (2018) employed a task that allowed objects to be expanded or compressed in depth, and found no systematic distortion along depth for binocularly viewed symmetric objects. However, their finding cannot be used as evidence for shape constancy because they did not manipulate viewing distance.

The research described in the present article was designed to fill this void in the literature. We investigated stereoscopic shape constancy using stimuli that satisfy the minimal conditions of Li et al. (2011) model with a response task that is sensitive to systematic variations in the relative apparent depths of objects. Observers were required to match a stereoscopic test object presented at different viewing distances by adjusting the depth-to-width ratio of a comparison object at a fixed viewing distance. The stimuli included both symmetric and asymmetric objects. The symmetric objects were similar to those used in Li et al. (2011) and satisfied all the required conditions for their model. The asymmetric objects, in contrast, violated both the symmetry and planarity constraints which are essential for the shape-from-symmetry computation. If the observers can use the 3D shape information provided by symmetry to circumvent the need for disparity scaling, they should achieve shape constancy when viewing symmetric objects, but fail to do so when viewing asymmetric objects. However, this is not what we found. The results revealed that there were large effects of viewing distance for both types of objects, and that the presence or absence of symmetry had no detectable effect on performance.

## Method

### Participants

Thirteen observers participated in the experiment, including the 3 authors and 10 others who were naïve about the purpose of the experiment. All the participants reported normal or corrected-to-normal visual acuity. All gave informed consent as approved by the Institutional Review Board at the Ohio State University.

### Stimulus Displays

Ten mirror-symmetric 3D polyhedra were used for the experiment, which were quite similar to those used by Li et al. (2011). They each had 16 vertices, connected by 14 quadrilateral faces with one mirror symmetry plane. The faces of each polyhedron were painted in three different colors such that no two adjacent faces were the same. All visible edges were rendered in white and hidden edges were removed.

**Figure 1.**
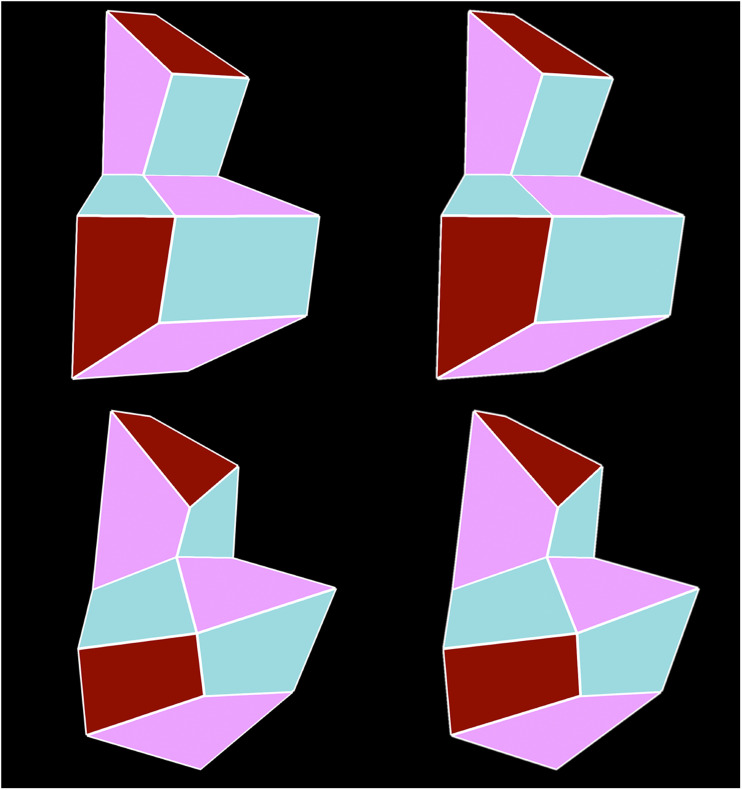
Two stereograms of a symmetric object (top) and an asymmetric object (bottom) that were used in the present experiment.

The asymmetric objects were created by displacing the vertices of the symmetric ones. Specifically, each of the 16 vertices of a symmetric polyhedron was randomly displaced by about 10% of the spatial extent of the original object along an arbitrary direction in 3D. Then the displaced vertices were connected in the same way as for the original object. Three such asymmetric objects were generated for each of the 10 symmetric polyhedra. Figure 1 shows stereograms of a symmetric object (top) and one of its asymmetric distortions (bottom). Note that some faces (e.g., the top and bottom faces) of the asymmetric object appear curved.

### Apparatus

The 3D stimuli were generated in Matlab in real time and rendered with PsychOpenGL, a set of essential functions that interfaces Psychtoolbox (Kleiner et al., 2007; Pelli, 1997) with OpenGL. For any given stimulus, two slightly different stereoscopic perspective images were computed for observers’ left and right eyes using a technique called horizontal image translation (Lipton, 1991) that horizontally shifts the viewpoint of each eye by an amount determined by the interocular distance measured for each observer. This produces an optically correct pattern of horizontal and vertical disparities. The observer viewed the stereoscopic images binocularly through LCD shutter glasses (NVIDIA 3D Vision 2) that were synchronized with the refresh rate of a mosaic display so that each eye received the appropriate image.

The mosaic display was composed of two identical LCD monitors (Dell S2716DG) placed side by side. They were synchronized into a unified and seamless display by NVIDIA Mosaic technology and bezel correction. The refresh rate of the mosaic display was 120 Hz. Thus, the image for each eye was updated at the rate of 60 Hz, which was fast enough to avoid flicker. The mosaic display had a horizontal and vertical extent of 120 × 34 cm, and its spatial resolution was 5160 × 1440 pixels. The observers viewed the display in a darkened room at a distance of 150 cm while using a chinrest to restrict head movements. However, they were free to move their eyes, which allowed them to fixate anywhere within the viewing space to achieve maximum acuity for objects presented in different locations.

### Procedure

**Figure 2.**
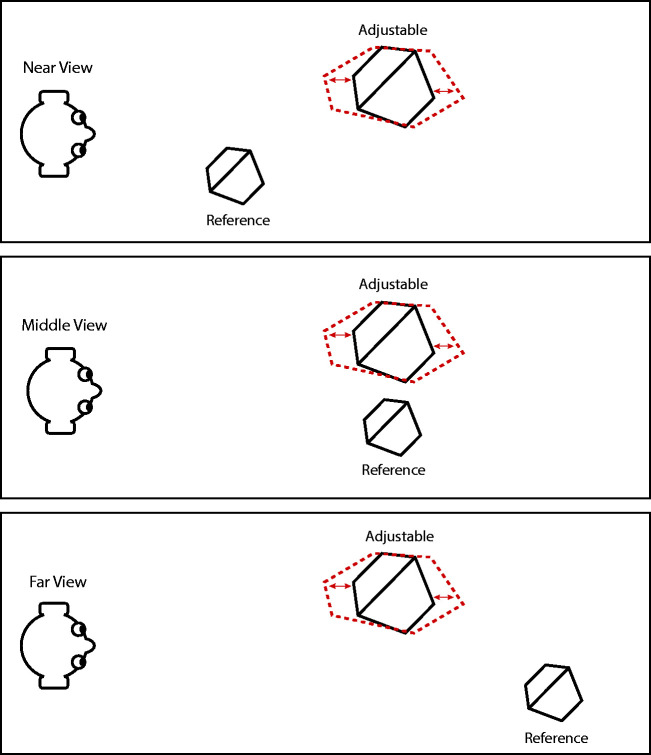
A bird’s eye view of the viewing geometries used in the present experiment. An adjustable object was always presented in the left hemifield at a distance of 1.5 m. The reference object was always presented in the right hemifield, and its simulated viewing distance was manipulated across trials with possible values of 0.7 m, 1.5 m, and 2.3 m. Observers were required to expand or compress the adjustable object in depth so that its apparent shape matched that of the reference object.

The basic scene geometry of the experiment is shown in Figure 2. Two stimulus objects were presented side by side against a black background on the mosaic display during each trial presentation. The horizontal distance between the rightmost vertex of the left object and the leftmost vertex of the right object was 9 cm and the central line between them was midway between the eyes. The objects were shown at eye level. The one on the right had a fixed 3D shape and we will refer to it as the *reference* object. The one on the left could be expanded or compressed in depth by the observer and it is referred to as the *adjustable* object. The two objects shown in each trial had identical 3D shapes except for their depth-to-width ratios. The initial depth-to-width ratio of the adjustable object was set randomly. Both objects were presented in the same 3D orientation. For the symmetric objects, the symmetry planes were at a 45° angle relative to the line of sight because that is the slant that is most favorable for structure-from-symmetry computations. The asymmetric objects were created by randomly displacing the vertices of the symmetric ones.

The observers’ task was to adjust the shape of the adjustable object by expanding or compressing it in depth using a handheld mouse so that it matched the apparent shape of the reference object. On trials in which the reference object was symmetric, the adjustable object was asymmetrically distorted in depth, except for one possible setting where it matched the shape of the reference object.

The simulated viewing distance to the adjustable object was always 150 cm, which was the same as the physical distance between the observer and the mosaic display. The simulated viewing distance of the reference object was manipulated across trials with three possible values of 70 cm, 150 cm, and 230 cm (see Figure 2). The two objects presented in each trial were rendered in different colors and sizes so that their 2D images were not identical. The size of the adjustable object was approximately the same on every trial. The average horizontal and vertical extents of the 10 possible objects were 10.9 cm and 16.8 cm, respectively. Its extension in depth could vary based on the observers’ settings. The physical size of the reference object was always set at 70% of the adjustable object and was fixed across different simulated viewing distances for a given polyhedron. As a result, the size of its 2D projected image would change with distance. The selection of objects was constrained so that no faces appeared or disappeared from view due to the adjustment.

The experiment was performed in a dark and quiet room where the display was the only source of illumination. Prior to their participation, observers were asked to perform several practice trials to get familiar with the equipment and the task. During these practice sessions all of the observers indicated that they could clearly perceive the compressions and expansions in depth of the adjustment object. The practice trials used a different object than the ones used in the experimental trials. At the start of each trial, the depth-to-width ratio of the adjustable object was set randomly. Observers then moved the mouse horizontally to make adjustments without time limitation.

Each observer conducted two separate sessions: one for the symmetric objects, and one for the asymmetric objects. The order of the two sessions was counterbalanced across observers. Within each session, three possible viewing distances were presented three times each for each of the ten polyhedral objects used in this experiment. Therefore, one session had 90 trials and was run in two blocks of 45 trials with randomized order. On average, one session took about 40 minutes.

## Results

During their debriefing sessions, all of the observers reported that the displays produced perceptually vivid impressions of 3D structure, and that manipulations of the mouse produced clear changes in the apparent depth scaling of the adjustable object. Because the adjustable and reference objects on any given trial always had different sizes, it would not be meaningful to directly compare their relative extensions in depth. Thus, in order to normalize the size differences, we instead compared the depth-to-width ratio of the adjustable object relative to the depth-to-width ratio of the reference object. This can be expressed by the ratio:

$$S=\frac{z_{\mathrm{adj}}/x_{\mathrm{adj}}}{z_{\mathrm{ref}}/x_{\mathrm{ref}}}$$

where $z_{\mathrm{adj}}$ and $x_{\mathrm{adj}}$ represent the adjustable object’s extents along the Z-axis and X-axis, respectively, and $z_{\mathrm{ref}}$ and $x_{\mathrm{ref}}$ represent the extents of the reference object. Note that $z_{\mathrm{adj}}$ is the only variable that was controlled by the observers. Because this particular measure produces an imbalance between under- and over-estimates of an object’s extension in depth, we transformed the scale by using the binary logarithm of *S*. We will refer to this measure as the *log relative aspect ratio*. log_2_ *S* = 0 indicates a perfect shape match (up to a similarity transformation). log_2_ *S >* 0 indicates the adjustable object was expanded in depth, and log_2_ *S <* 0 indicates that the adjustable object was compressed in depth relative to the reference object.

**Figure 3.**
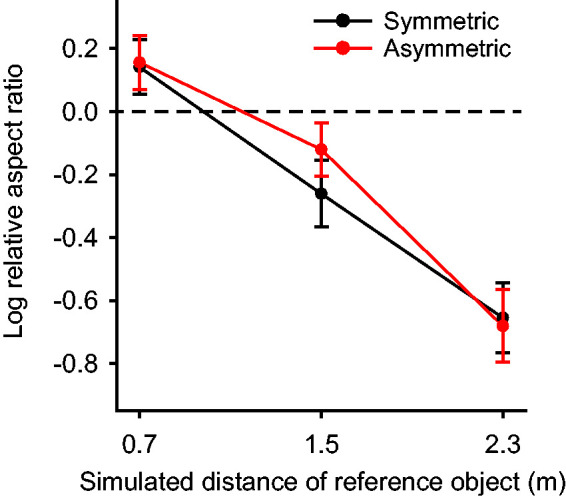
The average adjusted aspect ratio of 13 observers as a function of viewing distance for the symmetric and asymmetric conditions. The horizontal dashed line represents veridical performance. Error bars denote ±1 standard error of the mean.

Figure 3 shows the average responses over all observers plotted as a function of the simulated viewing distance for both symmetric and asymmetric objects. An analysis of variance (ANOVA) on the group data revealed a significant effect of viewing distance, *F*(2, 24) = 35.605, *p* < 10^−7^, but there was no significant effect of symmetry, *F*(1, 12) = 0.400, *p* = .539, and no significant interaction between distance and symmetry, *F*(2, 24) = 1.225, *p* = .312.

**Figure 4.**
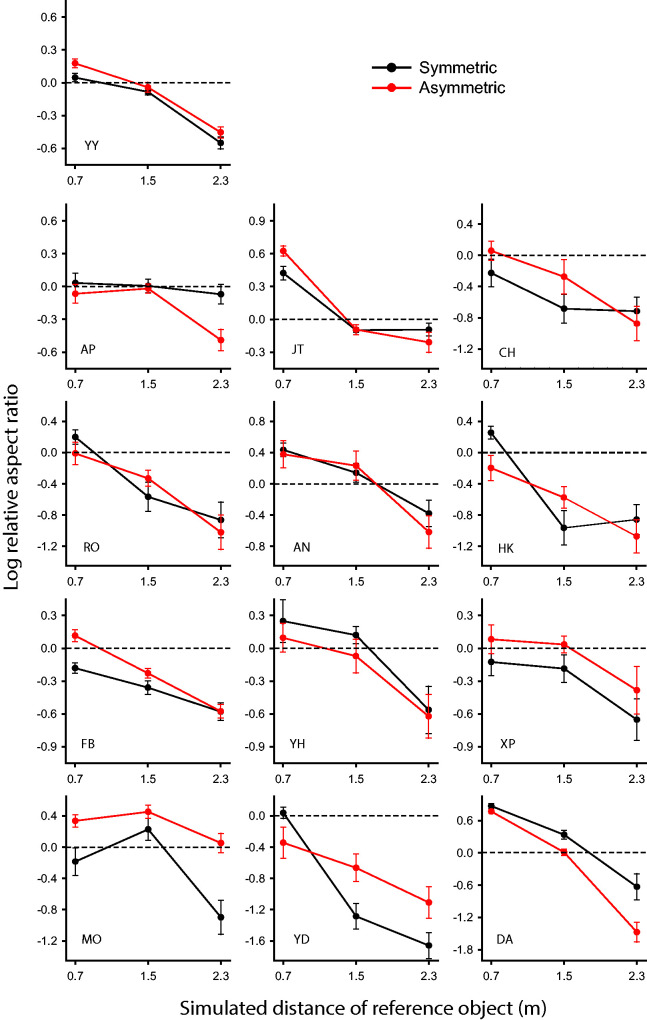
The adjusted aspect ratios of 13 individual observers as a function of viewing distance for the symmetric and asymmetric conditions. The horizontal dashed lines represent veridical performance. Error bars denote ± 1 standard error of the mean.

Figure 4 shows the individual data from all 13 observers. Analyses of variance revealed that each individual observer produced a significant effect of viewing distance, and all but three of those effects were at a .001 level of significance. It should also be noted, however, that there were substantial individual differences in the overall range of this effect, as has been reported in previous investigations (see Todd & Norman, 2003). For example, the judgments of AP, YY and FB covered a 0.6 range of log relative aspect ratios, whereas the ranges produced by MO, YD and DA were 3 to 4 times larger. At the closest viewing distance, most of the observers’ judgments closely matched the comparison object, but observers JT, YH, AN, and DA systematically overestimated the object depth in that case. Five of the observers had a significant effect of symmetry, though only two (DA and MO) reached the .01 level of significance. Interestingly, those two effects were in opposite directions, so they approximately canceled each other in the group analysis. Observer DA was the only one to produce a significant interaction between distance and symmetry.

Could the absence of any systematic effects of symmetry be due to a lack of experimental power? Additional analyses were performed in an effort to assess that issue. One approach involved the measurements of effect size (η^2^) for all of the different experimental manipulations (Levine & Hullett, 2002). The variations in simulated viewing distance accounted for 63% of the within subject variance (including error), whereas the combined effects of symmetry and the Symmetry × Distance interaction accounted for less than 1% of the variance. If there were a systematic effect of symmetry that remained undetected by this particular experimental design, it was over 60 times smaller in our sample than the robust effect of viewing distance that has been reported in many previous experiments.

A second approach was to employ a Bayesian version of a two-factor within-subject ANOVA (Rouder et al., 2017), using the statistical package developed by Morey and Rouder (2018). We considered three nested models within this framework: The simplest model (M1) included viewing Distance as the only fixed factor. The no-interaction model (M2) added Symmetry as a second fixed factor but allowed no Symmetry × Distance interaction. The most complex model (M3) included both factors and their interaction. All of these models also included participants as a random factor. We calculated Bayes factors (BFs) for the three pairwise comparisons among these models (Morey & Rouder, 2018; Rouder et al., 2017).

One advantage that Bayesian methods for model comparison have over traditional hypothesis-testing methods is that the former can be calibrated to reflect a reasonable set of beliefs informed by context and the literature (Rouder et al., 2017). This knowledge is injected into the mathematical model through the specification of priors. The technical details are deferred to the Appendix. Briefly, the three Bayesian ANOVA models are parameterized in terms of effect size—a dimensionless number similar to Cohen’s *d* (Cohen, 1992; not to be confused with the η^2^ measures in the frequentist analyses above). A separate effect size is estimated for each level of each experimental factor. The scale of the variability of effect sizes across levels within factors must be specified a priori and is an important part of the model specification. In the notation of Rouder et al. (2017), let *h* be the prior parameter that sets the scale of this variability. Different settings of *h* correspond to different substantive hypotheses.

We performed a series of Bayes-factor analyses for a range of settings of the scale parameter. First, we performed it with *h* = 0.5, which is the default value and specifies a preponderance of medium-sized effects for most levels of most factors (Rouder et al., 2017). For the comparison of Model 1 over Model 2, we obtained BF(1,2) ≈ 11. That is, the conditional probability of our data given a model (M1) that specifies no effect of Symmetry is 11 times greater than the conditional probability of the same data given a model (M2) that specifies a medium-sized effect of Symmetry. Note that any Bayes factor greater than 10 is generally interpreted as “strong” evidence in favor of one model over the other (Jeffreys, 1961). Thus, we have strong evidence in favor of no effect over a medium-sized main effect of Symmetry. For the comparison of Model 2 over Model 3, we obtained BF(2,3) ≈ 18. Based on analogous considerations, we interpret this as strong evidence in favor of no Symmetry × Distance interaction over a medium-sized interaction effect. The two Bayes factors can be multiplied to compare the simplest model (M1) to the most complex one (M3): BF(1,3) = BF(1,2) × BF(2,3) ≈ 200. Thus, we have “decisive” evidence in favor of the simplest model (Jeffreys, 1961). Overall, our data rule out the hypothesis that symmetry has a medium-sized effect on shape constancy—either by itself or in interaction with viewing distance.

What about *small-sized* effects of symmetry? Setting the scale parameter *h* to a smaller value allows us to explore this possibility. Concretely, we used *h* = 0.2, which is the lowest “good choice” for this parameter according to Rouder et al. (2017, p. 310). This setting specifies a preponderance of (very) small effects for most levels of most factors. This reduces the contrast among the three competing models and, consequently, brings the Bayes factors closer to 1. In our data set, we obtained BF(1,2) ≈ 4.7 for the first and BF(2,3) ≈ 3.9 for the second comparison. Although not as strong, these values still constitute “substantial” evidence in favor of the simpler model in each pair (Jeffreys, 1961). Their product, BF(1,3) ≈ 18, provides “strong” evidence in favor of the simplest model over the most complex one. Overall, our data cast serious doubt on the hypothesis that symmetry has even a small-sized effect on shape constancy. Note that this result is compatible with the traditional ANOVA reported above, in which symmetry accounted for just a negligible portion of the total variance. A more complex model always (over)fits the data better than a simpler nested model, but in the present case the improvement is too small (less than 1% of the variance) to warrant the inclusion of additional parameters.

For completeness, we repeated the calculations for *h* = 1.0, which is the highest “good choice” for the scale parameter (Rouder et al., 2017). This setting specifies a preponderance of (very) large effects. All Bayes factors became more extreme, as expected: BF(1,2) ≈ 22, BF(2,3) ≈ 70, and BF(1,3) ≈ 1500. Thus, our data decisively rule out large effects of symmetry.

The descriptive statistics about effect sizes reinforce the conclusions based on Bayes factors. The posterior estimates of effect-size for distance were practically invariant (up to the third decimal place) to the choice of model and the scale prior *h*. Concretely, *d*_0.7_ = 0.383, *d*_1.5_ = 0.046, and *d*_2.3_= –0.429, where the subscript indicates the distance in meters. The 95% highest-density credible interval (HDCI) around each estimate had an average half-width of ±0.052. The residual variance was $\sigma^{2}=0.831\;\pm \;0.048$ for the simplest model M1 with default *h* = 0.5. See Equation 1 in the Appendix for details. The inclusion of Symmetry and Symmetry × Distance interaction (model M3, *h* = 0.2) reduced this variance only by 0.0012. This echoes the result of the frequentist analysis where Symmetry had η^2^ < 0.01. The Bayesian effect-size estimates were *s*_1_ = –0.021for the symmetric and *s*_0_ = +0.021 for the asymmetric condition, HDCI = ±0.036. Finally, the effect-size estimates for the interaction were *u*_0.7_ = 0.012, *u*_1.5_ = –0.042, and *u*_2.3_ = 0.030 in the symmetric condition, HDCI = ±0.049; the values flipped signs in the asymmetric condition. Note that the latter two HDCIs included zero and were themselves contained in the interval [–0.1, 0.1].

## Discussion

There are several possible strategies by which it might be possible to achieve accurate performance on this task. For example, one traditional hypothesis is that observers use accommodation and/or convergence in order to scale disparities with viewing distance. Some researchers have argued that perceptual distortions can occur when using computer displays because accommodation and convergence provide conflicting information. When observing real objects in the natural environment there is a gradient of accommodative blur that occurs when different points on an object are located at different distances, especially in low illumination when the pupil diameter is relatively large. The absence of accommodative blur for computer displays provides conflicting information that the depicted objects are flat (see Watt et al., 2005). However, we would expect in that case that the conflict would be greatest at close viewing distances because that is where gradients of accommodative blur are most visible. This suggests that objects at near distances should appear compressed relative to those at far distances, which is the opposite of what is typically found in this type of experiment. Moreover, similar results have also been obtained for judgments of real objects, where there is no conflict between and accommodation and convergence (e.g., see Todd & Norman, 2003).

Another possible way of scaling stereopsis with viewing distance might be to exploit the vertical disparities between the projections on the two eyes. Previous mathematical analyses have shown that vertical disparities provide potential information for determining an object’s distance from the observer (Koenderink & van Doorn, 1976; Petrov, 1980), and there is empirical evidence to show that human observers are able to make use of that information in at least some contexts (Rogers & Bradshaw, 1993). However, an important limitation of vertical disparities is that they become vanishingly small at small visual angles, so that their effectiveness as a source of information may be restricted to objects with relatively large angular extents. This suggests that observers’ judgments of 3D shape from stereo should be most accurate for objects that are relatively large and/or relatively close to the point of observation, which is quite consistent with the pattern of results obtained in this experiment.

It is important to keep in mind that the apparent compression of objects in depth with increasing viewing distance is consistent with a large number of previous studies on stereoscopic shape constancy (e.g., Baird & Biersdorf, 1967; Campagnoli et al., 2017; Campagnoli & Domini, 2019; Gilinsky, 1951; Glennerster et al., 1996, 1998; Harway, 1963; Hecht et al., 1999; Heine, 1900; Johnston, 1991; Johnston et al., 1994; Norman et al., 1996; Scarfe & Hibbard, 2006; Shaffer et al., 2008; Todd & Norman, 2003; Toye, 1986; Wagner, 1985), although there are relatively large differences among these studies in the overall range of this effect. The primary purpose of this particular replication was to test a hypothesis by Li et al. (2011) that it is possible to obtain veridical judgments of 3D shape if visual information from binocular disparity is combined with additional computational constraints imposed by the presence of bilateral symmetry. The results of the present study provide no support for that hypothesis. The observers’ judgments of symmetric polyhedra were systematically affected by viewing distance, but the presence or absence of symmetry had no significant effect on these judgments (see also Phillips et al., 2011).

Before considering the overall implications of these findings, there are a couple of methodological issues that deserve to be highlighted. All of the observers in this experiment agreed that the response task was quite difficult. Although they could clearly perceive the expansions and contractions in depth of the adjustment stimulus, they were not completely certain about which setting best matched the shape of the reference object. It is well known in the 3D shape perception literature that observers will sometimes adopt short cuts for performing psychophysical judgments if they can rely on artifactual information (see Todd & Norman, 2003), and it is important to consider what short cuts might be available whenever one is designing a discrimination or matching task.

**Figure 5.**
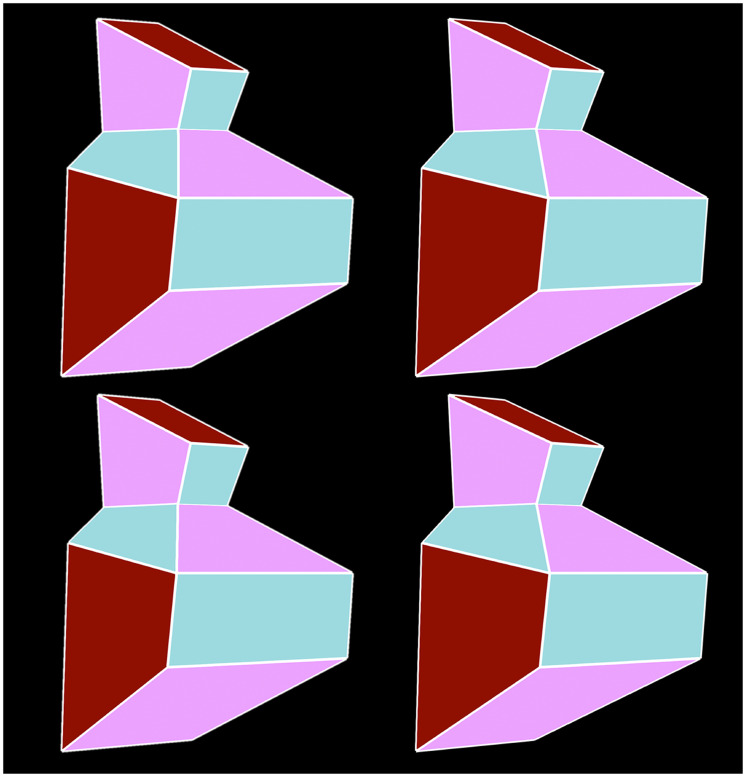
Two stereograms of a symmetric object and an asymmetric object. Both objects are identical except for their relative extensions in depth, which differ by 20%. Note that it is difficult to determine which one is symmetric and which one is not.

For example, one possible shortcut for symmetric stimuli in the present experiment might be to ignore the reference stimulus altogether and to manipulate the adjustable object so that it appears symmetric. This strategy could produce veridical performance in the symmetric condition because all possible adjustment settings are physically asymmetric except for the one that correctly matches the reference object. Of course, if observers had successfully employed that strategy, it could not have produced an effect of viewing distance because the distance to the adjustable object never changed. We have performed an earlier study to examine whether this response strategy is perceptually possible (Yu et al., 2017). Observers were presented with a single adjustable object and asked to manipulate its extension in depth so that it appeared to be symmetric. The results revealed large systematic errors and large individual differences. For example, Figure 5 shows two stereograms of polyhedra that are identical in all respects except that their relative extensions in depth differ by 20%. One is symmetric and the other is not, but it is quite difficult to determine which is which.

There is another type of adjustment task used by Jayadevan et al. (2018), Li et al. (2009), and Li et al. (2011) to investigate the perception of 3D shape from symmetry for which the problem of shortcuts is much more serious. In their procedure, observers are presented with a stationary polyhedron, and they must match the shape of it with an adjustable object that rotates continuously in depth over a full 360°. Although it is difficult to determine which object in Figure 5 is symmetric, it would be trivial to do so if the objects were observed rotating in depth over 360°. In the experiment by Li et al. (2011) all objects in the adjustment space were symmetric, but the correct setting was always the one that was maximally compact. This would allow observers to perform the task by ignoring the reference object altogether, and simply adjusting the rotating one to maximize its compactness. Indeed, Yu et al. (2021) were able to confirm the viability of this strategy by replicating the results of Li et al. (2011) in conditions where the adjustment object was presented by itself with no reference object to compare it to. In the light of this observation, any experiment that obtains veridical shape judgments with continuously rotating adjustment stimuli should be viewed with great suspicion. This is especially true when most of the observers are fully aware of how the reference objects were created, as was the case in the experiments by Li et al. If we exclude the data from adjustment tasks with stimuli rotating in depth over 360°, we are not aware of any compelling evidence that bilateral symmetry plays any role at all in the perception of 3D shape.

The big question that remains to be answered is why observers fail to exploit the possible information about 3D shape that is provided by bilateral symmetry. We believe the best way to understand this is to consider the limitations of models that have been proposed for the computation of 3D shape from symmetry. For example, the model developed by Li et al. (2011) can only produce veridical estimates of 3D shape if the observed object is a bilaterally symmetric plane-faced polyhedron with a sufficient level of complexity. It must also be presented over a limited range of orientations, have a maximum degree of compactness, and be viewed under orthographic projection. The probability of satisfying all those conditions simultaneously in the natural environment is quite small. Thus, the adaptive advantage of such a mechanism would most likely be of negligible value relative to the metabolic cost of maintaining specialized brain circuits for the special case of bilaterally symmetric, compact, complex, plane-faced polyhedra.

An alternative strategy that is well supported by empirical data is that observers’ stereoscopic shape judgments are based on patterns of binocular disparity with a less than perfect scaling parameter. This will produce perceptual errors relative to the ground truth that are highly constrained to expansions or contractions along the line of sight, but those errors would likely have no impact at all on any real world task an observer might need to perform in the natural environment. Readers may judge for themselves which of these strategies is more ecologically plausible.

## Appendix: Details of the Bayes-Factor Analysis

All Bayesian analyses were performed with the anovaBF function in the *BayesFactor* statistical package (Morey & Rouder, 2018; Rouder et al., 2017). We considered a combination of three models analogous to traditional within-subject ANOVA. The equation of the most complex model (designated M3) is

(1) $$\log_{2}S_{\mathrm{ijk}}=\mu +\sigma p_{k}+\sigma (d_{i}^{*}+s_{j}^{*}+u_{ij}^{*})+\epsilon_{\mathrm{ijk}}$$

where the dependent measure on the left-hand side is the log relative aspect ratio defined in the main text, μ is the grand mean, and $\epsilon_{\mathrm{ijk}}$ are independent, identically distributed, zero-centered Gaussian noise terms with variance $\sigma^{2}$. The index *i* ranges over the 3 levels of viewing Distance, *j* over the 2 levels of Symmetry, and *k* over the 13 participants in our sample. The no-interaction model M2 drops the term $u_{ij}^{*}$ for the Symmetry × Distance interaction, and the simplest model M1 also drops the main effect $s_{j}^{*}$ of Symmetry. Note that Equation 1 assumes homogeneity of variance—the standard-deviation parameter σ is common for all participants. Although the confidence intervals in Figure 4 indicate a clear violation of this assumption in our data, it is adopted in the *BayesFactor* software (and in traditional ANOVA) because it simplifies the calculations considerably. The individual means, by contrast, are accommodated by the random effects *p*_k_ of participants. All within-participant factors are treated as fixed effects—a sum-to-zero constraint is imposed on each respective set of coefficients. This is denoted by the asterisks in Equation 1 and is implemented by multiplying each set of coefficients by a matrix of suitably chosen rank (see Rouder et al., 2012, for details). Thus, for example, the 6 interaction terms $u_{ij}^{*}$ have only 2 effective degrees of freedom.

A key innovation relative to traditional ANOVA is the re-parameterization in terms of effect sizes instead of unscaled deviations from the grand mean (Rouder et al., 2012, 2017). Because the treatment effects are scaled by the standard deviation σ in Equation 1, they are dimensionless ratios that can be interpreted regardless of the units of the dependent variable. They are closely related to a commonly used measure of effect size, Cohen’s *d* (Cohen, 1992). Crucially, this unit-invariance allows the placement of proper informative priors on the effect-size parameters (Jeffreys, 1961). The anovaBF models use a hierarchical approach in which the scale of the effect sizes is determined by a variance parameter separately for each factor

(2) $$d_{i}^{*}|\;g_{d}∼\mathrm{Normal}(0,g_{d}),\;\;i=0.7,\;1.5,\;2.3s_{j}^{*}|\;g_{s}∼\mathrm{Normal}(0,g_{s}),\;\;\;j=0,1u_{ij}^{*}|\;g_{u}∼\mathrm{Normal}(0,g_{u}),\;\;i=0.7,1.5,2.3;\;j=0\text{,}1$$

and a (hyper)parameter *h* specifies the scale of the variance parameters themselves

(3) $$g_{d}∼\mathrm{Inv}\chi^{2}\left(1,h\right)\;g_{s}∼\mathrm{Inv}\chi^{2}\left(1,h\right)\;g_{u}∼\mathrm{Inv}\chi^{2}(1,h)$$

where $\mathrm{Inv}\chi^{2}$ denotes the scaled inverse chi-square distribution with a single degree of freedom. To generate a draw *g* from this distribution, first draw ${X∼\chi }^{2}(1)$ and then let *g* = *h/X*. Equation 3 uses a common scale *h* for all treatment factors for simplicity. A separate parameter *h*_p_ sets the scale of the random Participant factor (Equation 4). It was fixed at its default *h*_p_ = 1 in all analyses. The grand mean μ and residual variance $\sigma^{2}$ had noninformative priors (Rouder et al., 2017).

(4) $$p_{k}|\;g_{p}∼\mathrm{Normal}\left(0,g_{p}\right),\;k=1,2,\ldots ,13\;\;g_{p}∼\mathrm{Inv}\chi^{2}(1,h_{p})$$

The scale parameter *h* must be set a priori, and different settings specify different substantive hypotheses about the magnitude of typical effect sizes. Thus, it is important to calibrate this parameter with reference to typical effect sizes reported in the relevant literature (Rosenthal & DiMatteo, 2001). We refer the reader to Figure 4 in Rouder et al. (2017), which plots the marginal distribution of effect sizes for various settings of *h*. According to Rouder et al. (2017, p. 310), good choices are 0.2 < *h* < 1, with a default of 0.5. For comparison, many psychological studies report Cohen’s *d* in the same range, with 0.5 conventionally designated as a “medium-sized” effect (Cohen, 1992). This numerical comparability stems from the scaling of $\mathrm{Inv}\chi^{2}$ in Equation 3. Still, *h* and *d* are separate variables that are only stochastically related (Equation 2), and hence *h* should not be interpreted uncritically in terms of Cohen’s (1992) conventional scale for *d*. The null hypothesis of no effect corresponds to the limit h→0.

It is useful to address briefly two potential methodological concerns. First, some researchers prefer noninformative to informative priors because the former are considered less subjective. They may be tempted to choose a large value for *h* in an effort to avoid commitment to any particular effect size. This would be misguided in this context because very large values of *h* specify a definite commitment to very large effect sizes, thereby diluting the marginal likelihood of small- and medium-sized effects (Rouder et al., 2017; Wagenmakers et al., 2010). Second, some methodologists (e.g., Kruschke, 2015) have expressed concerns that Bayes factors are too sensitive to the choice of priors. Indeed, this can become a problem if the priors are specified in an ad hoc manner. These methodologists recommend a posterior-estimation approach instead, on the grounds that posterior estimates are less prior-sensitive for many models (though not all). For example, Kruschke (2015, Chapter 12) recommends estimating highest-density credible intervals (HDCIs) for the parameters of interest—effect sizes in our case—and then comparing these HDCIs to a pre-established region of practical equivalence (ROPE). In our opinion, the differences between the posterior-estimation and Bayes-factor approaches to statistical inference are less important than their commonalities. We advance two arguments—one mathematical and one conceptual—in support of this claim. Mathematically, the two approaches can be unified with a certain model specification, now popular in the statistical literature, called *spike-and-slab* priors (Rouder et al., 2018). Conceptually, both approaches agree that evidence for the null hypothesis should be calibrated against a belief in how big effect sizes are expected to be if the null were false. In the posterior-estimation approach, this information is contained in the ROPE, whose bounds are determined by prior domain knowledge and a review of the literature. In the Bayes-factor approach, equivalent information is supplied by the parameter *h* that scales the variability of effect sizes (Equation 3). The prior setting for *h* is based on exactly the same sources. We considered an explicit range of “good choices” reported in the main text.

The Bayes factors throughout this article are reported with two-digit precision because it is limited by Monte Carlo fluctuations. The descriptive statistics about the effect sizes are based on 100,000 samples from the posterior distribution of models M1 and M3. The posterior distribution of any given effect-size parameter (e.g., *s*_0_ in Equation 1) was symmetric and unimodal. Consequently, the mean, median, mode, and the halfway point of the 95% HDCI coincided to three decimal places. They were also nearly invariant with respect to the choice of model and of the scale prior *h*. This allowed the use of a simple notation in the Results section. For example, *s*_0_ = 0.021 ± 0.036 denotes that the posterior mean, median, and mode of *s*_0_ were all equal to 0.021, and the 95% HDCI had a half-width of 0.036 and extended symmetrically on either side of the point estimate.
